# Phenotype, molecular characterisation and risk factors for postoperative meningitis caused by ESBL-producing-*Enterobacteriaceae*: a six years multi-Centre comparative cohort study

**DOI:** 10.1186/s12879-021-05784-7

**Published:** 2021-01-19

**Authors:** Guanghui Zheng, Yanfei Cao, Chunhong Liu, Lingye Qian, Yumeng Cai, Miaomiao Cui, Huiting Sun, Lv Hong, Jun Yuan, Lina Zhang, Guojun Zhang

**Affiliations:** 1 Department of Clinical Diagnosis, Laboratory of Beijing Tiantan Hospital and Capital Medical University, NO. 119 Nansihuan West road, Fengtai district, Beijing, China; 2 Daqing Oilfield General Hospital Clinical Laboratory, No. 9 Zhongkang Street, Saltu District, Daqing, China; 3 Department of Clinical Diagnosis, Laboratory of Sanbo Brain Hospital and Capital Medical University, NO.50 Yikesong Road, Haidian District, Beijing, China; 4 Department of Clinical Diagnosis, Laboratory of the Second People’s Hospital of Guiyang, Guiyang, China

**Keywords:** ESBL, *Enterobacteriaceae*, Meningitis, Molecular characterisation, Risk factor

## Abstract

**Background:**

To determine the phenotype, molecular characterisation and risk factors of postoperative meningitis induced by Extended-spectrum β-lactamase (ESBL)-producing *Enterobacteriaceae* (EPE) in China.

**Methods:**

We performed a multi-centre comparative cohort study of postoperative meningitis patients infected with *Enterobacteriaceae* in 4 neurosurgical centres in China from January 2014 to December 2019. Phenotype and molecular characteristics of the isolates were reviewed and tested, and independent risk factors of the EPE meningitis were evaluated by binary logistic regression.

**Results:**

In total, 220 *Enterobacteriaceae* include 78 EPE were available in this study. 85.6% (67/78) ESBL-related genes were tested, and *bla*_*SHV*_ (14.9%) and *bla*_*SHV*_ + *bla*_*TEM*_ + *bla*_*CTX-M-9*_ (20.9%) were found to be the most frequent mono and combined ESBL-related genes harboured by *Enterobacteriaceae.* On binary logistic analysis, craniotomy (OR. 2.583, 95% C.I. 1.274–5.235, *P* = 0.008) and malignancy (OR. 2.406, 95% C.I. 1.299–4.456, *P* = 0.005) were the associated independent risk factors to meningitis induced by EPE.

**Conclusions:**

To the best of our knowledge, this is the largest series focusing on risk factors of EPE meningitis which has been conducted in China. Craniotomy and malignancy were independent risk factors for EPE meningitis. The risk factors identified may be further utilized in clinical practice and research to avoid and reduce the mortality in future.

## Background

Postoperative meningitis, secondary to neurosurgical procedures, trauma, or shunt devices, is one of common healthcare-associated infections, which are a significant cause of perioperative morbidity, mortality and health-care cost [1, 2]. Incidence of postoperative meningitis in recent prospective studies varied between 0.7 and 25% [3–6]. In recent years, postoperative meningitis has been found to be closely related to the success of neurosurgical operation and mortality of neurosurgery patients; therefore, it has received increasing attention in clinical settings. Patients who underwent neurosurgery are known to have lower immunity, experience complicated longer duration of operations, and are more difficult to treat [7]. Therefore, reducing the incidence of postoperative meningitis is an important task for neurosurgical physicians.

Various pathogens can cause postoperative meningitis, of which, and *Enterobacteriaceae* is a critical branch. *Enterobacteriaceae* has been reported to account for more than 20% of nosocomial infections [8]. Multidrug-resistant *Enterobacteriaceae* are more harmful due to their extensive drug resistance. Extended-spectrum β-lactamase (ESBLs) production is one the most common antibiotic resistance mechanism of *Enterobacteriaceae*, and ESBL-producing *Enterobacteriaceae* (EPE) has been an increasingly implicated as a cause of infection. A previously study reported that prevalence of EPE rectal colonisation in healthy human beings was 14% globally, and the distribution varied extremely in different locations [9]. Also, EPE, as a nosocomial pathogen, is a health threat among medical institutions, especially in surgery patients with immune-compromised or other comorbidities. In 2017, the WHO released a list of antibiotic-resistant bacteria that pose the greatest threat to human health, and for which new antibiotics are desperately needed. From that, EPE belongs to the first-grade priority (critical) and is a clinically ultra-threatening pathogen [10]. The risk factors for EPE include prolonged mechanical ventilation [11], ICU admission [12], catheter usage [13], severe illness [14], and frequent antibiotic usage [15]. However, few reports are target on risk assessment of postoperative meningitis. Existence of the blood-brain barrier may block antibiotics such as polymyxin B entering the central nervous system, and lead to EPE postoperative meningitis more harmful [16], therefore, risk factor assessment possess great clinical significance.

To better prevent and determine the treatment strategy of postoperative meningitis caused by EPE, we conducted a multi-centre comparative cohort study to explore the independent risk factors and assess clinical molecular characteristics of postoperative meningitis caused by EPE. To our knowledge, this is the first study globally to assess the molecular characteristics of EPE and risk factors of EPE postoperative meningitis.

## Methods

### Study design

A comparative cohort study was performed at four neurological centres in China, including Beijing Tiantan Hospital and Capital Medical University, Sanbo Brain Hospital and Capital Medical University, Daqing Oilfield General Hospital and The Second People’s Hospital of Guiyang between Jan 2014 and Dec 2019. The EPE molecular characteristics test of this study was approved by the ethical committee of Beijing Tiantan Hospital and Capital Medical University (KY-2019-095-03).

### Patients

**Fig. 1:**
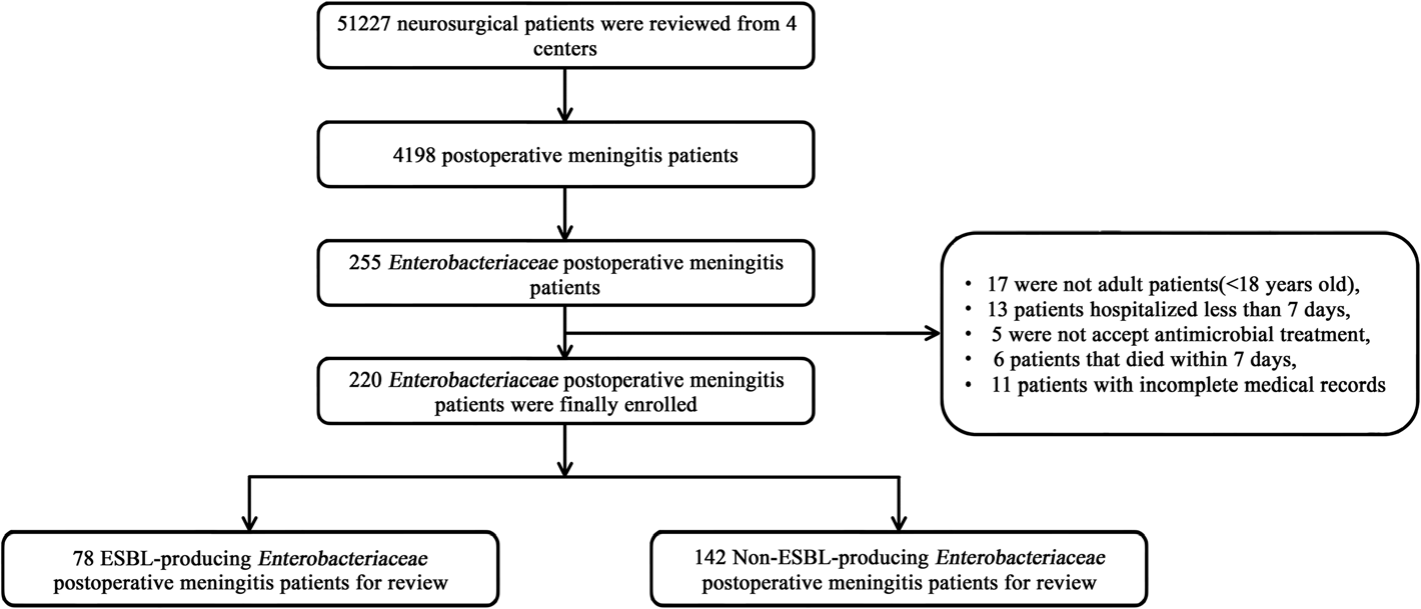
Flow chart of the selection of *Enterobacteriaceae* postoperative meningitis patients

In the four centres, adult neurosurgical patients(> 18 years old) were included if they were survived at least 7 days with at least one cerebrospinal fluid (CSF) culture positive for *Enterobacteriaceae*. Patients who underwent only external ventricular drain (EVD) or CSF shunt or stereotactic surgery, without antimicrobial therapy not done in hospital and incomplete clinical medical records were excluded. All of the patients were followed-up for the diagnosis of postoperative meningitis during the first 30 days after neurosurgery. A flow chart of this study is shown in Fig. 1.

Patients with *Enterobacteriaceae* postoperative meningitis were diagnosed by the diagnostic criteria of the Centres for Disease Control [17, 18]. The criteria of *Enterobacteriaceae* meningitis was as follows: 1) *Enterobacteriaceae* cultured from neurosurgical patients’ CSF; 2) one or more of the clinical symptoms or signs, including fever(> 38 °C), headache, meningeal signs (stiff neck, cranial nerve signs or irritability), with anti-infective treatment; and one or more of the clinical laboratory tests as follows: 1) elevated CSF protein level, increased CSF leucocyte count, and/or decreased CSF glucose level; 2) appearance of gram-negative bacillus on gram stain of CSF; 3) growth of *Enterobacteriaceae* in patients’ blood (by bacterial culture).

### Microbiology

All bacteria with the same growth characteristics of *Enterobacteriaceae* were classified to standard microbial identification procedure, and the identification system is VITEK-2 Compact system ((bioMerieux, Marcy l ‘etoile, France, based on biochemical reaction) and VITEK MS (bioMerieux, Marcy l ‘etoile, France, based on matrix-assisted laser desorption/ionization time-of-flight mass spectrometry) system. Antimicrobial testing of susceptibility (AST) of *Enterobacteriaceae* was performed by the disc diffusion tests (Kirby-Bauer method) and broth microdilution method (MIC) and classified as sensitive, intermediate and resistant according to the Clinical and Laboratory Standards Institute (CLSI) 2019. Isolates tested with aztreonam or ceftazidime MICs > 1 mg/L were screened for the presence of ESBL. Judgment of an ESBL phenotype of four *Enterobacteriaceae (Klebsiella pneumoniae, Klebsiella oxytoca, Escherichia coli* and *Proteus mirabilis)* was performed by Kirby-Bauer method according to the CLSI 2019. The test procedure is as follows: ceftazidime/clavulanate and cefotaxime/clavulanate discs, in comparison with ceftazidime and cefotaxime discs alone (Oxoid Ltd., Basingstoke, Unite Kingdom). According to the CLSI 2019 antimicrobial susceptibility testing standards [19], EPE was defined when an increase in a zone diameter ≥ 5 mm for either antibiotic together with clavulanate vs the zone diameter of the agent, and it is reported that the ESBL phenotype of other *Enterobacteriaceae* can also be determined by the same method as the above four *Enterobacteriaceae* [20]. The left were defined as non-ESBL-producing *Enterobacteriaceae* (NEPE).

Molecular detection of *bla*_*CTX-M-1*_*, bla*_*CTX-M-9*_*, bla*_*SHV*_*, bla*_*TEM*_*, bla*_*OXA-23*_ *and bla*_*OXA-66*_ were done by micro/nanofluidic chip platform (MNCP) based on the loop-mediated isothermal amplification (LAMP) method [21]. The extraction of the nucleic acid was done according to MNCP manufacturer’s instructions. In addition, preparation of the isothermal amplification reaction solution and detection using the MNCP were the same as previously described [22].

### Therapy and clinical variables for evaluation

In this study, all of the patients’ qualified daily progress records were established by analyse of the clinical database of the neurosurgery, infectious diseases and microbiology departments in the four centres described above. From that, we summarised and measured the status of antibiotic use and clinical outcomes in patients with postoperative meningitis, including antibiotic prophylaxis, empirical, definitive therapy and mortality. Antibiotics were employed by the neurosurgical doctors according to local or international common standards and antimicrobial susceptibility testing. Twenty-one characteristics of the postoperative meningitis patients were extracted from the clinical database for risk factor evaluation, including patients’ routine information (age, male%), fever(> 38 °C), assist mechanical ventilation (AMV), bacteraemia, craniotomy, CSF leakage, diabetes mellitus, EVD, Glasgow Coma Scale (GCS), hospital-acquired pneumonia, hypertension, intensive care unit (ICU) admission, lumbar drainage (LD), long-time surgery duration(> 180 min), length of hospital stay (LOS), malignancy, postoperative infection time, reoperation, surgical wound classification, and time of cure of infection.

### Statistical analysis

All of the variables differences were evaluated by univariate analysis. Of them, the categorical variables were assessed by Pearson’s chi-squared test. Quantitative data were assessed using Student’s t-test or one-way variance analysis test, and abnormally distributed quantitative variables were processed using Kruskal-Wallis or Mann-Whitney *U* test. Binary logistic algorithm was built to evaluate differences between the EPE and NEPE postoperative meningitis patients. Any variables with *P* < 0.1 in the univariate analysis were carried forward in binary logistic regression algorithm to analyse the independent risk factor for EPE postoperative meningitis. Significance was defined as a *P* < 0.05, and calibration was analysed by Hosmere Lemeshow (H-L) test for goodness-of-fit. Statistical analyses were carried out by SPSS 22.0 (IBM, New York, USA). The graph was performed using Prism 7.0 (Graphpad, San Diego, USA).

## Result

### Patients

Over the 6 years of the study, a total of 51,227 neurosurgery patients and 4198 postoperative meningitis patients were included in the four centres described above. The infection rate was 8.2% (4198/51,227). Two hundred seventy-two cases of *Enterobacteriaceae* postoperative meningitis were recorded. Of them, 52 were excluded based on criteria (Fig. 1).

### Microbiology

**Fig. 2:**
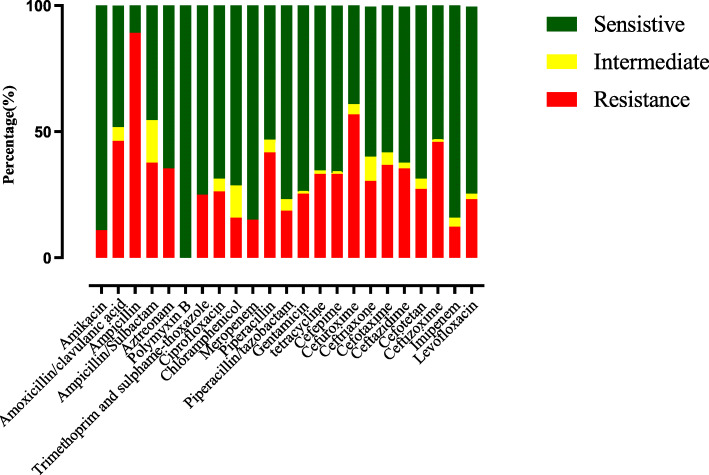
Antimicrobial susceptibility test of *Enterobacteriaceae* enrolled in four neurosurgical centres

**Fig. 3:**
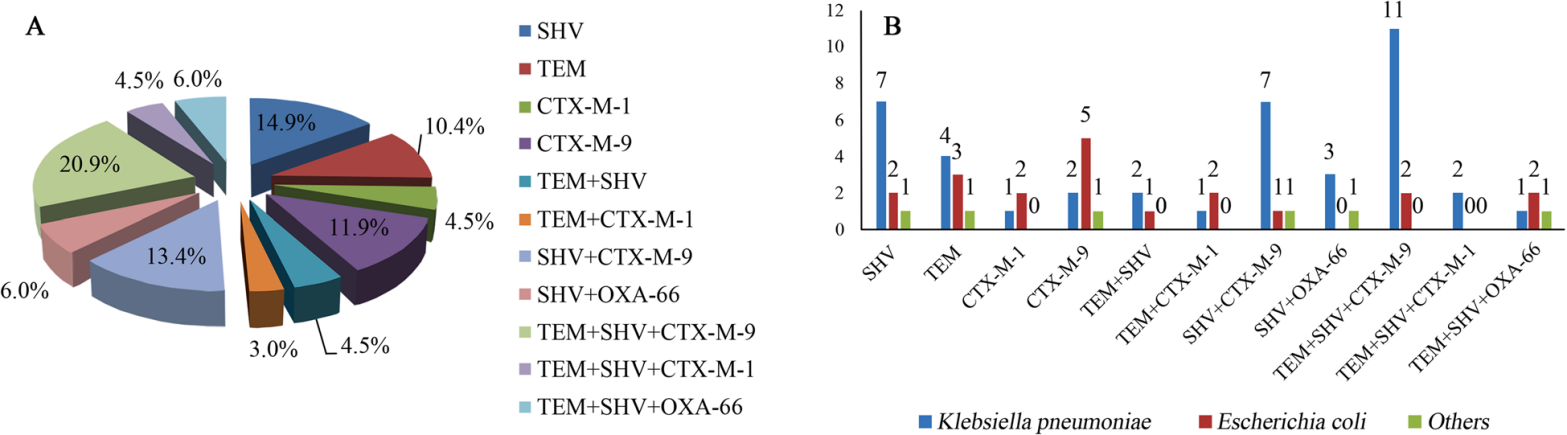
Distribution of ESBL-related genes among the EPE isolates (**a**). Distribution of ESBL types carried by *K. pneumoniae*, *E. coli* and other EPE isolates (**b**) collected from 2014 to 2019 in four neurosurgical centres in China

The distribution of *Enterobacteriaceae* is shown in Table 1. From that, *Klebsiella pneumoniae* has the highest proportion (40.9%, 90/220), followed by *Escherichia coli* (17.3%, 38/220), *Enterobacter aerogenes* (10.0%, 22/220), and *Enterobacter cloacae* (9.1%, 20/220*)*. The antimicrobial susceptibility test of the *Enterobacteriaceae* is shown in Fig. 2. The proportion of EPE was 35.5%. In carbapenem, sensitivity to meropenem and imipenem were both 85.0%. Among the 78 EPE, 67 isolates of ESBL-related genes were detected by MNCP, and the *bla*_*SHV*_ gene is the most frequent ESBL-related gene. Majority (58.2%, 39/67) of the gene-harbouring EPE contained multiple genes, and the most frequent ESBL-related gene combination of EPE is *bla*_*SHV*_ + *bla*_*TEM*_ + *bla*_*CTX-M-9*_. The whole distribution of the genes is shown in Fig. 3-A. Also, we classified all of the 67 EPE into 3 groups, including *K. pneumonia*, *E. coli* and others. The numbers of each groups’ genes are shown in Fig. 3-B.

**Table 1:** Distribution of *Enterobacteriaceae* Species in four neurosurgical centres

*Enterobacteriaceae*	EPE(78)	NEPE(142)	Total(220)
*Citrobacter koseri*	0 (0.0%)	4 (2.8%)	4 (1.8%)
*Enterobacter aerogenes*	6 (7.7%)	16 (11.3%)	22 (10.0%)
*Enterobacter cloacae*	4 (5.1%)	16 (11.3%)	20 (9.1%)
*Enterobacter gergoviae*	0 (0.0%)	1 (0.7%)	1 (0.4%)
*Enterobacter hormaechei*	0 (0.0%)	2 (1.4%)	2 (0.9%)
*Enterobacter sakazakii*	0 (0.0%)	1 (0.7%)	1 (0.4%)
*Escherichia coli*	20 (25.6%)	18 (12.7%)	38 (17.3%)
*Klebsiella oxytoca*	0 (0.0%)	11 (7.8%)	11 (5.0%)
*Klebsiella pneumoniae*	42 (53.8%)	48 (33.8%)	90 (40.9%)
*Morganella morganii*	0 (0.0%)	1 (0.7%)	1 (0.4%)
*Pantoea agglomerans*	2 (2.6%)	7 (4.9%)	9 (4.1%)
*Proteus mirabilis*	1 (1.3%)	0 (0.0%)	1 (0.4%)
*Proteus rettgeri*	0 (0.0%)	2 (1.4%)	2 (0.9%)
*Serratia marcescens*	2 (2.6%)	15 (10.6%)	17 (7.7%)
*Serratia plymuthica*	1 (1.3%)	0 (0.0%)	1 (0.4%)

### Therapy and clinical outcomes of patients with Enterobacteriaceae postoperative meningitis

Table 2 shows clinical therapy and outcomes of patients with *Enterobacteriaceae* postoperative meningitis. Of them 95.5% (210/220), 94.6% (208/220) and 97.7% (215/220) of patients received antibiotic prophylaxis, empirical antibiotics medication and definitive therapy. Finally 83.6% (184/220) patients were cured in total, and 16.4% (36/220) patients died; among them, the mortality attributed to infection rate was 11.8% (26/220). There were no significant differences between the EPE and NEPE postoperative meningitis in the three treatment methods. However, in comparison with NEPE group, clinical outcome of mortality attribute to infection was significantly higher in EPE group (18.0% VS 8.4%, χ2 = 4.358, *P* = 0.049).

**Table 2:** Therapy and outcomes of EPE postoperative meningitis

Antibiotics	Total (220)	EPE (78)	NEPE (142)	χ2	*P*
**Antibiotic prophylaxis**	210 (95.4%)	73 (93.6%)	133 (93.7%)	–	0.999
Ceftazidime	13 (5.9%)	7 (9.0%)	6 (4.2%)	2.055	0.229
Ceftriaxone	34 (15.4%)	14 (18.0%)	20 (14.1%)	0.589	0.440
Cefuroxime	109 (49.6%)	38 (48.7%)	71 (50.0%)	0.596	0.460
Meropenem	9 (4.1%)	3 (3.8%)	6 (4.2%)	0.018	0.999
Erythromycin	13 (5.9%)	5 (6.4%)	8 (5.6%)	0.055	0.774
Cefoperazone/Sulbactam	11 (5.0%)	2 (2.6%)	9 (6.3%)	1.512	0.334
Others	17 (7.7%)	4 (5.1%)	13 (9.2%)	1.148	0.428
**Received empirical antibiotics**	208 (94.6%)	73 (93.6%)	135 (95.1%)	0.241	0.758
Single antibiotics	75 (34.1%)	23 (29.5%)	52 (36.6%)	1.010	0.365
Ceftriaxone	11 (5.0%)	4 (5.13%)	7 (4.9%)	0.197	0.728
Meropenem	46 (20.9%)	15 (19.2%)	31 (21.8%)	0.211	0.798
Cefuroxime	4 (1.8%)	1 (1.3%)	3 (2.1%)	0.064	0.999
Vancomycin	14 (6.4%)	3 (3.8%)	11 (7.8%)	0.691	0.529
Combination antibiotics (2)	113 (51.4%)	42 (53.8%)	71 (50.0%)	0.466	0.560
Vancomycin + Meropenem	96 (43.6%)	35 (44.9%)	61 (43.0%)	0.138	0.788
Vancomycin + Ceftazidime	7 (3.2%)	3 (3.8%)	4 (2.8%)	0.103	0.710
Others	10 (4.6%)	4 (5.1%)	6 (4.2%)	0.038	0.999
Combination antibiotics (3)	28 (12.7%)	9 (11.5%)	19 (13.4%)	0.124	0.833
Vancomycin + Meropenem +Cefuroxime	8 (3.6%)	3 (3.8%)	5 (3.5%)	0.147	0.999
Vancomycin + Meropenem +Tinidazole	6 (2.7%)	3 (3.8%)	3 (2.1%)	1.116	0.352
Others	14 (6.4%)	3 (3.8%)	11 (7.8%)	1.474	0.420
**Received definitive therapy**	215 (97.7%)	74 (94.9%)	141 (99.3%)	4.436	0.055
Single antibiotics	67 (30.4%)	19 (24.4%)	48 (33.8%)	1.584	0.220
Meropenem	61 (27.7%)	16 (20.5%)	45 (31.7%)	1.519	0.341
Ceftriaxone	4 (1.8%)	2 (2.6%)	2 (1.4%)	0.981	0.371
Colistin	2 (0.9%)	1 (1.3%)	1 (0.7%)	0.475	0.490
Combination antibiotics (2)	128 (58.2%)	48 (61.5%)	80 (56.3%)	1.331	0.306
Vancomycin + Meropenem	105 (47.7%)	39 (50.0%)	66 (46.5%)	0.032	0.999
Vancomycin + Ceftazidime	8 (3.6%)	3 (3.8%)	5 (3.5%)	0.001	0.999
Meropenem + Tigecycline	8 (3.6%)	2 (2.6%)	6 (4.2%)	0.569	0.709
Others	7 (3.2%)	4 (5.1%)	3 (2.1%)	1.219	0.424
Combination antibiotics (3)	20 (9.1%)	7 (9.0%)	13 (9.2%)	0.003	0.999
Colistin+ Meropenem + Trimethoprim	7 (3.2%)	2 (2.6%)	5 (3.5%)	0.196	0.999
Colistin+ Tigecycline + Meropenem	6 (2.7%)	3 (3.8%)	3 (2.1%)	0.848	0.613
Others	7 (3.2%)	2 (2.6%)	5 (3.5%)	0.196	0.999
**Clinical Outcome**					
Improved and cured	184 (83.6%)	62 (79.5%)	122 (85.9%)	1.520	0.254
Mortality attributed to infection	26 (11.8%)	14 (18.0%)	12 (8.4%)	4.358	0.049
Mortality not attributed to infection	10 (4.6%)	2 (2.6%)	8 (5.6%)	1.093	0.500

### Univariate analysis of risk factors for EPE postoperative meningitis

The main demographic and clinical characteristic data of EPE and NEPE postoperative meningitis groups are shown in Table 3. In univariate analysis of 21 factors in EPE- and NEPE-related postoperative meningitis patients, 4 factors were clinical significantly different (*P* < 0.05), including: AMV (χ2 = 0.007, *P* = 0.007), surgical wound classification (χ2 = 5.265, *P* = 0.024), craniotomy (χ2 = 14.675, *P* < 0.001) and malignancy (χ2 = 4.466, *P* = 0.048).

**Table 3:** Characteristics of patients and Univariate analysis between f EPE and NEPE postoperative meningitis

Characteristics	Total (220)	EPE (78)	NEPE (142)	Z/χ2	*P*
Age (years)				0.400	0.565
Median	45	45	43		
IQR	30–55	30–59	31–52		
Male%	126 (57.3%)	49 (62.8%)	77 (54.2%)	1.520	0.255
Hypertension	45 (20.4%)	18 (23.1%)	27 (19.0%)	0.511	0.489
Diabetes mellitus	16 (7.3%)	5 (6.4%)	11 (7.8%)	0.133	0.793
Fever (b.t > 38 °C)	130 (59.1%)	42 (53.8%)	88 (62.0%)	1.375	0.254
LD	99 (45.0%)	40 (51.3%)	59 (41.3%)	1.927	0.202
EVD	113 (51.4%)	36 (46.2%)	77 (54.2%)	1.313	0.263
Long surgery duration (> 180 min)	126 (57.3%)	50 (64.1%)	76 (53.5%)	2.304	0.155
CSF Leakage	48 (21.8%)	20 (25.6%)	28 (19.7%)	1.035	0.312
Reoperation	56 (25.4%)	24 (30.8%)	32 (22.5%)	1.799	0.198
AMV	72 (32.7%)	35 (44.9%)	37 (26.1%)	8.095	0.007
LOS (days)				1.326	0.198
Median	35	38	34		
IQR	20–41	20–42	20–40		
Time of cure of infection (days)				−1.620	0.626
Median	13	13	13		
IQR	8–20	7–18	8–22		
Surgical wound classification				5.265	0.024
Clean (I)	116 (52.7%)	51 (65.4%)	70 (49.3%)		
Clean-contaminate (II)	104 (47.3%)	27 (34.6%)	72 (51.7%)		
Craniotomy	114 (51.8%)	54 (69.2%)	60 (42.2%)	14.675	< 0.001
GCS				−1.984	0.232
Median	8	8	9		
IQR	6–12	5–10	7–12		
Postoperative infection time				1.284	0.366
Median	7	8	7		
IQR	4–13	4–14	3–12		
ICU admission	89 (40.4%)	37 (47.4%)	52 (36.6%)	2.445	0.151
Malignancy	103 (46.8%)	44 (56.4%)	59 (41.6%)	4.466	0.048
Bacteraemia	49 (22.3%)	19 (24.4%)	30 (21.1%)	0.304	0.613
Hospital-acquired pneumonia	67 (30.4%)	27 (34.6%)	40 (28.2%)	0.988	0.359

### Binary logistic analysis of risk factors for EPE postoperative meningitis

We conducted a binary logistic analysis to evaluate the independent risk factor of EPE postoperative meningitis (Table 4). Firstly, we included all the factors with *P* < 0.1 in the univariate analysis into the binary logistic analysis. Then, the H-L test of the model was calculated for calibration. All the variables embedded in the algorithm by binary logistic analysis were listed in Table 4. From that, craniotomy (odds ratio (OR) 2.583, 95% confidence interval(C.I.) 1.274–5.235, *P* = 0.008) and malignancy (OR 2.406, 95% C.I. 1.299–4.456, *P* = 0.005) were independent risk factors for EPE meningitis, and the H-L test is 0.773 (> 0.05).

**Table 4:** Binary logistic analysis of risk factors for EPE postoperative meningitis (OR: odds ratio; C.I.: confidence interval; H-L: Hosmere Lemeshow)

Variables	*P*	OR	95% C.I.	H-L test
Surgical wound classification	0.703	1.134	0.594–2.169	0.773
Craniotomy	0.008	2.583	1.274–5.235	
Malignancy	0.005	2.406	1.299–4.456	
AMV	0.123	1.652	0.873–3.127	

## Discussion

In this multi-centre study, we conduct the largest neurosurgical individuals with *Enterobacteriaceae* postoperative meningitis up to now, including 220 adult patients during 2014–2019. Characteristics of the molecular epidemiology, and risk factors in EPE postoperative meningitis were screened. Of them, craniotomy (OR 2.583, 95% C.I. 1.274–5.235, *P* = 0.008) and malignancy (OR 2.406, 95% C.I. 1.299–4.456, *P* = 0.005) were independent risk factors for EPE postoperative meningitis, and the mortality attributed to infection between the two groups is significantly different. It was also confirmed that the majority of EPE contained more than one ESBL related gene, while *bla*_*SHV*_ + *bla*_*TEM*_ + *bla*_*CTX-M-9*_ is the most frequent gene combination.

*Enterobacteriaceae* is one of the critical pathogenic bacteria that cause nosocomial infections, occupying a large proportion of all pathogenic bacteria. As previously reported, EPE has played a vital role in nosocomial infection [23]. It has the properties of easy to acquire resistance genes (e.g., horizontal gene transfer) [24], and high pathogenicity [25]. It has strong drug resistance characteristics, and the incidence of ESBL production is 35.5%, but the proportion of ESBL production by *K. pneumoniae* and *E. coli* is as high as 46.7 and 52.6%. The WHO ranks EPE as the first priority of grade (critical), it has extensive drug resistance and is resistant to most β-lactam antibiotics except carbapenem. High rates of sensitivity were found against aminoglycoside; however, ototoxicity and nephrotoxicity of the aminoglycoside blocked clinical applications [26].

This study confirmed that the majority of EPEs does not pose single ESBL-resistance gene. The proportion of EPE with a single gene is 41.8%. Among them, the *bla*_*SHV*_ gene is the most frequent ESBL-related gene harboured by *Enterobacteriaceae* strains in the four centres of China during the past 6 years. ESBL-related genes are distributed differently around the world. Similar to our findings, one report conducted by Yahaya al [27] shows that *bla*_*SHV*_ (36.4%) was the most frequent genotype in EPE, followed by *bla*_*TEM*_ (31.4%) and *bla*_*CTX-M*_ (27.3%). In a previous analysis of ESBL carriage of *Enterobacteriaceae*, *bla*_*CTX-M-15*_ was the dominant ESBL-producing gene in all European countries except Greece, where *bla*_*SHV*_ were more common [28]. In the Netherlands, it’s reported that *bla*_*CTX-M-1*_ was the predominant gene [29]. Previously studies above certified that predominance gene varied in different regions and probably determine the resistance phenotypes of the microorganisms. Further, the dominant genotypes of distinct EPE are different; For example, the highest genotype proportion of *K. pneumoniae* is *bla*_*SHV*_, and that of *E. coli* is *bla*_*CTX-M-15*_. Meanwhile, it was reported that EPE always carried various ESBL-related genes other than mono-genotype [30]. The most frequent ESBL-related gene combination of EPE is *bla*_*SHV*_ + *bla*_*TEM*_ + *bla*_*CTX-M-9*_(20.9%), followed by *bla*_*SHV*_ + *bla*_*CTX-M-9*_(13.4%), and the possible reason as follows: Firstly, plasmids possessing *bla*_CTX-M_ genotypes are admitted to possessing other ESBL-related genes transmitting resistance to a series of antibiotics. Secondly, single replicon in distinct resistance gene location may lead to co-selection and may contribute to the dissemination [31].

The second- or third-generation cephalosporins were employed as antibiotic prophylaxis for postoperative meningitis patients ahead of the neurosurgery, which can prevent the invasion of pathogenic bacteria during the operation. In the empirical treatment, there are more antibiotics for selection, but vancomycin+meropenem is the main choice. This is because neurosurgery is generally complicated, and the use of the most effective antibiotics, such as carbarpenem, can most likely reduce the infection rate. In the definitive therapy, small percentage of patient population received monotherapy with meropenem. Carbarpenem seems to be the most frequently used antibiotic to cure EPE infections. Practice guidelines for management of ventriculitis/meningitis have been announced previously [32], a third-generation generation cephalosporin, such as ceftriaxone is endorsed for NEPE meningitis, while meropenem is endorsed as the first-line drugs target on EPE meningitis by general guidelines [33, 34].

Nevertheless, carbapenems should be used with caution because its overuse will generate acquisition of carbarpenem intermediate/resistant isolates by degrees. Also, resistance to third-generation cephalosporin in *Enterobacteriaceae* is always associated with ESBL, whereas resistance to carbapenems can be caused by production of an ESBL or plasmid mediated coded AmpC cephalosporinase combined with an efflux pump system or a decrease in outer membrane permeability [35–37]. It is reported that the mutation rate of the membrane permeability of *Enterobacteriaceae* is relatively high [38, 39], so when dealing with meningitis caused by EPE, even if it is treated with carbapenem antibiotics, good clinical outcomes may not be obtained. All of the 220 cases of *Enterobacteriaceae* in this study were sensitive to polymyxin B, which maybe the last line of defence against EPE or Carbapenem-resistant *Enterobacteriaceae* (CRE). It is reported that polymyxin B can be injected intrathecally in patients with severe Gram-negative bacteria [40], which maybe the ultimate choice for *Enterobacteriaceae* infection. However, polymyxin B causes nephrotoxicity, neuromuscular blockages and other adverse reactions, such as respiratory depression, and its application is limited to patients with critical disease. Therefore, to better deal with EPE postoperative meningitis, prevention of infection is still one of the main choices.

At present, several studies have explored the risk factors of postoperative meningitis. However, most of them focused on the incidence and total risk factors of neurosurgical meningitis. For example, a case-control study reports that patients with EVD, LD and diabetes were risk factors of meningitis after neurosurgery [3]. Another study showed that, hydrocephalus, Koos grade IV, operative duration > 3 h and intraoperative bleeding volume >400 ml were significantly were independent risk factors of postoperative meningitis after microsurgery for vestibular schwannoma. Fewer studies have focused on clinical characteristics and risk factors, specifically for multi-drug resistant bacterial meningitis. Pintado et al. conducted a comparative cohort study to evaluate the prognostic factors of methicillin-resistant *Staphylococcus aureus* meningitis and concluded that mortality was related to indwell of cerebrospinal devices (OR 7.9, 95% CI 3.1–20.3, *P* < 0.001) [2]. Yagel conducted a matched case-control study to evaluate the bacteraemia caused by EPE and NEPE, and the result showed that Gram-negative bacteria have statistically significantly different factors involved in successful and failed treatment, including pathogen types, highest body temperature in the first 24 h of symptoms, CSF glucose content and meropenem susceptibility [41]. In this study, 21 variables were evaluated and binary logistic analysis of risk factors indicating that craniotomy and malignancy are individual independent risk factors of EPE postoperative meningitis. For craniotomy and malignancy patients, surgical operation is complicated, and the prognosis and the patient’s own immunity is poor, which may lead to a high incidence of infection in patients. In addition, patients with malignancy have adopted high-level preoperative and empirical treatment [42], which may induce resistance to *Enterobacteriaceae*. Also, due to poor patient prognosis, the proportion of patients who gradually received high-grade antibiotics is high, and NEPE that cannot infect the patient’s central nervous system directly may lead to *Enterobacteriaceae* meningitis.

Some limitations still exist in this study. First, this is a retrospective study, and the conclusion mostly depends on the accuracy of the data in hospital, which may result in selection bias, although we did a prospective genetic study of *Enterobacteriaceae*. Second, we did not include all clinical variables related to meningitis, such as grade of the tumors, multiple catheter insertion, and primary clinical laboratory tests were not embedded. In our next study, we will conduct a prospective study to include more neurosurgical centres (6–8) and patients (300–500) and strive to achieve a universal risk factor assessment algorithm across China, creating a more objective risk factor assessment and prediction of EPE postoperative meningitis.

## Conclusion

To our knowledge, this study is the first one to conduct a multi-centre molecular and comparative cohort study of EPE postoperative meningitis. *Enterobacteriaceae* postoperative meningitis is a relatively serious clinical challenge worldwide. We have determined the characteristics of the *Enterobacteriaceae*, molecular epidemiology and evaluated the risk factors and treatment of postoperative meningitis. Craniotomy and malignancy were determined to be independent risk factors for EPE postoperative meningitis, and it is necessary to pay attention to prevention and treatment clinically.

## Data Availability

The datasets used and/or analysed during this study are available from the corresponding author on reasonable request.

## Abbreviations

EPE: ESBL-producing *Enterobacteriaceae*
CSF: cerebrospinal fluid
EVD: external ventricular drain
MIC: microdilution method
CLSI: Clinical and Laboratory Standards Institute
NEPE: non-ESBL-producing *Enterobacteriaceae*
MNCP: micro/nanofluidic chip platform
GCS: Glasgow Coma Scale
ICU: intensive care unit
LD: lumbar drainage
LOS: length of hospital stay
AMV: assist mechanical ventilation
H-L: Hosmere Lemeshow
